# An overview of cybercrime law in South Africa

**DOI:** 10.1365/s43439-023-00089-8

**Published:** 2023-06-02

**Authors:** Sizwe Snail ka Mtuze, Melody Musoni

**Affiliations:** Nelson Mandela University, Gqeberha, South Africa

**Keywords:** Unlawfull access of data, Malicious communications, Cyber Fraud

## Abstract

The COVID-19 pandemic has accelerated the uptake and use of information communication technologies and led to the digital transformation of different sectors of the economy. For South Africa, the COVID-19 pandemic struck at a time when the South African government had committed itself to leveraging technology for the benefit of its citizens, the private sector, and the public sector. By 2020, South Africa already had in place enabling policy and legal frameworks to assist with the regulation of activities taking place in cyberspace. The increase in broadband access has resulted in the increase of internet users. Due to increase in use of digital technologies and processing of personal data, there has been an increase in cyber-attacks and cybercrimes such as data breaches, identity theft and cyber fraud. Several South African based companies, state owned entities, government departments and citizens have been victims of cyber-attacks. To respond to the growing spectre of cybercrime, the South African government promulgated laws to supplement the existing legal framework. It also operationalised some of the laws which had been passed but had not yet come into operation. This paper gives a summary of the evolution of cybercrime laws in South Africa. It starts off by summarising how common law and the Electronic Communications and Transactions Act addressed cybercrime. The paper then proceeds to discuss the recently promulgated Cybercrimes Act, which is now the primary law criminalising certain online activities. It explores how the various provisions of the Cybercrimes Act address different types of cybercrimes known today. This discussion is aimed at demonstrating that South Africa is no longer a safe haven for cybercriminals.

## Cybercrime in South Africa

The COVID-19 pandemic has accelerated the uptake and use of information communication technologies and led to the digital transformation of different sectors of the economy. For South Africa, the COVID-19 pandemic struck at a time when the South African government had committed itself to leveraging technology for the benefit of its citizens, the private sector, and the public sector. By 2020, South Africa already had in place enabling policy and legal frameworks to assist with the regulation of activities taking place in cyberspace. The increase in broadband access has resulted in the increase of internet users. Due to increase in use of digital technologies and processing of personal data, there has been an increase in cyber-attacks and cybercrimes such as data breaches, identity theft and cyber fraud. Several South African based companies, state owned entities, government departments and citizens have been victims of cyber-attacks. To respond to the growing spectre of cybercrime, the South African government promulgated laws to supplement the existing legal framework. It also operationalised some of the laws which had been passed but had not yet come into operation.

This paper gives a summary of the evolution of cybercrime laws in South Africa. It starts off by summarising how common law and the Electronic Communications and Transactions Act addressed cybercrime. The paper then proceeds to discuss the recently promulgated Cybercrimes Act, which is now the primary law criminalising certain online activities. It explores how the various provisions of the Cybercrimes Act address different types of cybercrimes known today. This discussion is aimed at demonstrating that South Africa is no longer a safe haven for cybercriminals. On the 1st of June 2021, the President of the Republic of South Africa signed the Cybercrimes Act^1^ into law. This is the law which criminalises unlawful activities taking place in cyberspace. The President signed a Presidential Minute indicating that the commencement date of the whole Cybercrimes Act, except for certain sections, was the 1st of December 2021.^2^^,^^3^ These sections are discussed further below. Prior to the adoption of the Cybercrimes Act, some forms of cybercrime were criminalised either in terms of the common law or applicable statutory law such as the Electronic Communications and Transactions Act^4^.

Cronje et al. defined cybercrime as any criminal activity that involves a computer and includes crimes which previously existed before computers but now committed in a cyber environment such as fraud or child pornography^5^ and crimes which became possible because of the computer such as hacking, cracking and sniffing.^6^ Cassim notes the absence of a precise definition for computer crime but points out that in some instances the computer may be an object of a crime (for example when the computer itself is stolen) and in others, the subject of the crime (for example, when the computer is used to commit crimes such as fraud). Watney clarifies that a cybercrime may be committed either on the internet (for example website defacement) or on a computer which is not connected to the internet (for example deleting data).^7^

## Cybercrime under common law

Before legislation was enacted to criminalise certain unlawful cyber conduct, common law principles used to be extended as widely as possible to cater for the arrest and successful prosecution of online offenders.^8^ Snail argued that in certain instances, common law could be applied to conduct in cyberspace. For example, the offence of malicious damage to property could be committed in instances where a perpetrator disseminated a virus, trojan horse or worm into a computer system.^9^ Similarly, common law principles could be applicable to crimes such as defamation, child pornography, fraud, forgery, cyber smearing, if they are taking place online.^10^

One of the prominent cases which extended principles of common law to a cybercrime offence was the case of *S v Howard.*^11^ The court had to decide whether the accused committed the common law crime of malicious damage to property when malicious code loaded by him onto a computer network system belonging to his employer, Edgars Consolidated Stores Ltd, caused the erasure of intangible data. The Edgars store suffered losses of between 19 million and 57 million Rand. The question before the court was whether malicious injury to property could be committed where the property was not corporeal. The court held that temporary damage was done to corporeal property consisting of an integral unit of intangible software and tangible hardware. In this case, the court held that the crime of malicious damage to property was committed as the conduct by the accused caused an entire information system to break down.

Though common law could be used to criminalise certain online conduct, its application was limited and narrow.^12^ Over time, common law proved that it was not sufficient and adequate to address the innovative ways of committing crime. Common law revolves around the principle of *nullum crimen sine lege*, which means there is no crime without a law.^13^ The rationale or basis of the principle of legality are the policy considerations that the rules of criminal law ought to be as clear and precise as possible so that people may find out with reasonable ease and in advance how to behave to avoid committing crimes.^14^ South Africa has no codified common law principles. Instead of a criminal code, a large collection of authoritative decisions lays down the requirements for every common law crime as well as general criminal law principles.^15^

In terms of the common law principle of *ius acceptum,* a court may not find a person guilty of a crime unless the type of conduct he performed is recognised by the law as a crime.^16^ At the same time, a court may not create new crimes. In South Africa, the *ius acceptum* is understood to denote not only common law but also existing statutory law. The *ius acceptum* is anchored in South African law by virtue of section 35 (3) (l) of the Constitution.^17^

The other important common law principle is the *ius strictum*. In terms of this principle, crime creating provisions in both acts of parliament and subordinate legislation must be interpreted strictly.^18^ Snyman notes that ‘this principle of legality does not mean that a court should so slavishly adhere to the letter of the old sources of the law that common law crimes are deprived of playing a meaningful role in our modern society—a society which in many respects differs fundamentally from the society of centuries ago in which our common law writers lived’.^19^ The *ius strictum* principle implies further that a court is not authorised to extend a crime’s field of application by means of analogy to the detriment of the accused.^20^ This is also provided in terms of Article 22 (2) of the Rome Statute which provides that ‘the definition of a crime shall be strictly construed and shall not be extended by analogy. In case of ambiguity, the definition shall be interpreted in favour of the person being investigated, prosecuted, or convicted’.

Considering the limitations placed by the principles of legality, common law was not adequate to address novel cybercrime conduct. Earlier case law illustrated the need for specific legislation to address cybercrimes. Common law was ineffective in dealing with crimes such as spamming and phishing.^21^ Similarly, crimes such as website defacement, denial of service attacks or distributed denial of service attacks could not be adequately prosecuted by applying common law principles. As such, it became imperative for the legislature to introduce a law to address cybercrimes. The first law introduced to deal with cybercrimes was the Electronic Communications and Transactions Act.

## Cybercrime under the Electronic Communications and Transactions Act

As technology improved over the years, new ways for committing crime and new forms of crime started to emerge. One of the disadvantages brought by the rapid digitalization during the Fourth Industrial Revolution (4IR) was an increase in various cyber-attacks and offences such as cyber fraud, extortion, as well as forgery, malicious damage to property in the form of computer viruses, child pornography, hacking, cracking, and various other online activities.^22^ While common law could address some of these crimes, it was clear that a statutory instrument had to be put in place to regulate unlawful conduct in cyberspace. This led the legislature to promulgate a new law on cybercrimes. In 2002, the Electronic Communications and Transactions Act (ECT Act) was promulgated. It should be noted that apart from the ECT Act, there were other statutory instruments which addressed certain aspects of cybercrime.^23^ For instance, child pornography was criminalised in terms of the Films and Publications Act 65 of 1996, organized crime was prosecuted under the Prevention of Organised Crimes Act 121 of 1998 or the Financial Intelligence Centre Act 38 of 2001.

Chapter 13 of the ECT Act dealt with cybercrime, sections 85 to Section 90. Section 85 of the ECT provided for the regulation of unauthorized access to data without intentionally gaining access thereto but remaining. Section 86 of the ECT Act further regulates unauthorised access, interception of, or interference with data or denial of service attack. Van der Merwe et al. state that since the ECT Act came into force, the accused in a number of Regional Court cases have been successfully prosecuted for illegal accessing of data interms of Section 86 (1) of the ECT Act.^24^ In *R v Douvenga*, the accused was found guilty of contravening section 86 (1) of the ECT Act in that she intentionally and unlawfully gained entry to data which she knew contained confidential databases and emailed the data to her fiancée.^25^ Other court judgements where section 86 of the ECT Act was applied include the case of *Okundu v S*^26^, *Mgoqi v S*^27^ and *Myeni v S *in addition some of the perpetrators would also be charged with having contravened section 86 (2) of the ECT which deals with instances of data interferences which may also have amounted to a denial of service attack in terms of section 86 (5) of the ECT Act.^28^ In the case of *Salzmann v S*,^29^ the Court found that an offence in terms of section 86 (5) of the ECT Act is a serious one. It was argued that 86 (3) of the ECT Act introduced a new form of crime known as anti-cracking (anti-thwarting) and hacking law,^30^ which also criminalised being in possession of certain devices and their prohibited use as per section 86 (4) of the ECT Act.^31^

Section 87 of the ECT Act also deals with computer-related extortion, fraud and forgery. Attempt, aiding and abetting a cybercrime was also dealt with by section 88 of the ECT Act. Section 89 of the ECT Act set out the penalties for persons convicted in relation to provisions of the ECT Act.^32^ Section 89 of the ECT Act has been criticized for not being stringent enough to deter cybercriminals.^33^ For example, the maximum prison time for section 86 crimes was 12 months and for crimes such as fraud, extortion and forgery was a fine and imprisonment not exceeding 5 years.^34^ The legislature addressed this criticism and introduced stringent penalties under the Cybercrimes Act. For example, crimes of unlawful interception of data and unlawful interference with data or computer programs attract penalties of a fine or imprisonment for a period not exceeding ten years or both a fine and imprisonment.^35^

For nearly two decades, the ECT Act was the primary legislation criminalising cybercrimes. The courts have relied on different provisions of chapter 13 of the ECT Act to prosecute and convict offenders.

As can be imagined, the type of technologies which existed when the ECT Act was promulgated and the technologies in present day have drastically evolved. In the 4IR, there is extensive use of artificial intelligence, machine learning, cloud computing technologies, blockchain technologies and other disruptive technologies which did not exist over a decade ago. It became imperative for lawmakers in South Africa to introduce new laws which met these technological changes.

South Africa signed the Budapest Convention on Cyber Crime (ETS-185) in 2001 but never ratified it. It is unfortunate that South Africa has not yet ratified the Budapest Convention as the Convention provides solutions to law enforcement agents seeking to access remote cross border evidence. For instance, the recently signed 2nd Additional Protocol to the Budapest Convention addresses the jurisdictional challenges of cloud evidence and provides practical solutions to Member States. With most cloud data from South Africa being hosted in Europe, it is recommended for South Africa to ratify the Budapest Convention to enjoy the benefits of being a Member State.

Harmonisation of legal frameworks in Africa is also important to combat cybercrime and facilitate international cooperation.^36^ The African Union enacted the African Union Cyber Security and Protection of Personal Data Convention in 2014^37^(Malabo Convention). Chapter 3 of the Malabo Convention promotes cyber security and combating cybercrime. For the Malabo Convention to come into operation, it requires 15 Member States to ratify it. Currently, only 13 Member States have ratified the Convention, not including South Africa. It is commended that South Africa should ratify the Malabo Convention to make it easier for it to cooperate with law enforcement agents from other Member States. Furthermore, the Malabo Convention specifically calls for legislation against cybercrime by stating the following:

“Each State Party shall adopt such legislative and/or regulatory measures as it deems effective by considering as substantive criminal offences acts which affect the confidentiality, integrity, availability and survival of information and communication technology systems, the data they prove and the underlying network infrastructure, as well as effective procedural measures to pursue and prosecute offenders. State parties shall take into consideration the choice of language that is used in international best practices.”^38^

Furthermore, also in relation to the harmonization of domestic Cyber Crime regulation, the Malabo Convention states that:

“Each Member State shall ensure that the legislative measures adopted in respect of substantive and procedural provisions on Cyber Crime reflect international best practices and integrate the minimum standards contained in extant legislations in the region … to enhance the possibility of regional harmonization of the said legal measures.”

The Malabo Convention initially drew some important differences and proposed amendment to existing law with regards to attacks on computer systems, procedural law, attacks on computerized data, content related offenses, proposes adapting certain sanctions to the information and communication technologies, offenses relating to electronic message security measures, offenses specific to information and communication technologies and proposes adapting certain information and communication technologies offenses.^39^

The Cybercrimes Act was passed in 2021 as the law which would address the modern-day challenges that faced the criminal justice system considering both the Budapest Convention and Malabo Convention. The Cybercrimes Act repealed sections 85, 86, 87 and 88 of the ECT Act. It also substituted section 89 of the ECT Act by prescribing stricter penalties as mentioned above. The Cybercrimes Act is now a key regulation in creating offences and penalties for cybercriminality.^40^

## Cybercrime under the cybercrimes act

### An overview

The Cybercrimes Act expands offences under the ECT Act and criminalises more activities relating to the unlawful use of computer systems. It consists of nine chapters. Chapter 1 is the definitions and interpretation section. Chapter 2 addresses different forms of cybercrimes, malicious communications, sentencing and orders to protect complainants from the harmful effect of malicious communications. Chapter 3 pertains to issues of jurisdiction. Chapter 4 sets out the powers of law enforcement to investigate, search, access or seize. Chapter 5 contains provisions for mutual assistance between South Africa and foreign states. Chapter 6 contains provisions for the establishment and functions of a ‘designated point of contact’. Chapter 7 provides for the adducing of evidence by way of sworn statements. Chapter 8 sets out the obligations of electronic communications service providers and financial institutions to report cybercrime offences and preserving any information which may help regarding an investigation. Chapter 9 contains general provisions including the authority of the executive office to enter into agreements; the repeal and amendment of certain laws; the inclusion of regulations; and the commencement of the Act.^41^

The preamble of the Cybercrimes Act states that its purpose entails the creation of offences which have a bearing on cybercrime and to prescribe penalties for such crimes.^42^ The Cybercrimes Act makes amendments to eleven critical pieces of legislation. These are the Criminal Procedure Act 51 of 1977, the South African Police Services Act 68 of 1995, the Films and Publications Act 65 of 1996, the Criminal Law Amendment Act 105 of 1997, the National Prosecuting Authority Act 32 of 1998, the Correctional Services Act 111 of 1998, the Financial Intelligence Centre Act 38 of 2001, the Electronic Communications and Transactions Act 25 of 2002, the Regulation of Interception of Communications and Provision of Communication Related Information Act 70 of 2002, the Criminal Law (Sexual Offences and Related Matters) Amendment Act 32 of 2007 and the Child Justice Act 75 of 2008.

### Offences against confidentiality, availability and integrity of data

Part I of chapter 2 of the Cybercrimes Act addresses the different types of offences characterized as cybercrimes. The Cybercrimes Act does not specifically define the term ‘cybercrimes’.

Some apply the transformational test to categorise ‘cybercrimes’ into three generations. The first-generation crimes are cyber assisted crimes^43^, which pre-date the existence of computers, the internet and cyberspace.^44^ They include crimes such as murder, theft, robbery or “salami fraud”^45^ and the use of the computer is an incidental aspect of the commission of the crime and may afford evidence of the crime.^46^ Wall argues that if the internet were to be removed from the equation, these activities will still persist and be conducted through alternative forms of communication such as telephone and postal services.^47^ The second generation cybercrimes have a global scope through the advent of computer networks^48^ and without the internet, these crimes could continue to exist, but their scale would be drastically reduced.^49^ These crimes include dissemination of child abuse images^50^, child pornography, stalking, criminal copyright infringement and fraud.^51^ These crimes can be committed without computers, but the computer is used as a tool to commit the crime. The third generation consists of ‘true’ cybercrimes or computer dependent crimes.^52^ With these crimes, the computer or computer network is the target of the criminal activity.^53^ True cybercrimes include crimes such as hacking, denial of service attacks (DoS), distributed denial of service attacks (DDoS), website defacement and dissemination of malicious software.^54^

Offences criminalised as cybercrimes under the Cybercrimes Act include unlawful access (section 2), unlawful interception of data (section 3), unlawful acts in respect of software or hardware tools (section 4), unlawful interference with data or computer programs (section 5), unlawful interference with computer data storage mediums or computer systems (section 6), unlawful acquisition, possession, provision, receipt or use of a password, access code or similar data or device (section 7), cyber fraud (section 8), cyber forgery and uttering (section 9), cyber extortion (section 10) and theft of incorporeal property (section 12).

#### Unlawful access

The Cybercrimes Act prescribes two types of offences which can be committed under section 2. First, a person commits a cybercrime if he or she unlawfully and intentionally acts in respect of a computer system or computer data storage medium which places that person or any other person in a position to commit an offence.^55^ Secondly, section 2 also prohibits unlawful access to a computer system or a computer data storage medium.^56^ The section clarifies that a person accesses a computer data storage medium when data or a computer program stored on a computer data storage medium or store data or a computer program on a computer data storage medium is used.^57^ The section further clarifies that a person accesses a computer system if this person uses data or a computer program held in a computer system, or stores data or a computer program on a computer data storage medium forming part of the computer system or instructs, communications with, or otherwise uses, the computer system.^58^ The section describes that a person uses a computer program if he or she is in a position to copy or move the computer program to a different location, cause a computer program to perform any function, or obtain the output of a computer program.^59^ A person uses data if this person copies or moves the data to a different location or obtains the output of data.^60^

Unlawful access is normally referred to as hacking. Hacking is defined as gaining unauthorised access to a computer system, programs, or data.^61^ Hackers sometimes crack into government or business networks for profit or just for bragging.^62^ Different branches of the South African government have been subject to hacking attacks. The South African Police Services (SAPS) was hacked and criminals released details of thousands of whistle-blowers and victims.^63^ The State’s Government Communication and Information System was also compromised.^64^ The provisions of section 2 of the Cybercrimes Act criminalises anyone who may use any hacking software tools such as eBlaster^65^ to gain access to an organisation’s bank account for illicit and illegal purposes such as syphoning funds.^66^

#### Unlawful interception

Section 3 of the Cybercrimes Act creates an offence if an unlawful and intentional interception of data occurs. The author Snail gives examples of an unlawful interception of data to include a form of wiretapping, installing a sniffer to monitor communications on a network, and packet sniffing.^67^ Snail refers to Watney’s definition for surveillance as ‘to watch over’ and monitoring as ‘the listening to and/or reading of the content of communication’.^68^ It is proposed that since the term ‘monitoring’ is not specifically defined in the Cybercrimes Act, the definition from the Regulation of Interception of Communications and Provision of Communication-Related Information Act^69^ (RICA Act) should be considered.^70^ The RICA Act defines the term ‘monitor’ as ‘includes to listen to or record communication by means of a monitoring device’.^71^

Section 3 of the Cybercrimes Act prohibits three types of conduct. First, any unlawful and intentional interception of data.^72^ Interception of data can occur in instances where a cybercriminal clones a Visa debit card and intercepted data which was encoded on the magnetic strip of the debit card.^73^ Secondly, any unlawful and intentional possession of data or output of data knowing that such data was intercepted is prohibited.^74^ This would cover anyone who might not have committed the initial crime of interception but who is now in possession of the intercepted data and has the knowledge that the data under possession was illegally obtained. Thirdly, anyone found in possession of data or the output of data, regarding cases in which there is a reasonable suspicion that such data was intercepted unlawfully, and the person is unable to give satisfactory exculpatory account of such possession.^75^ Due to the volatility of data, it is quite possible for third parties to come into possession of data which has been unlawfully intercepted. It seems that the legislature added this criminal provision to prevent any further access and use of such data. If a third party is found in possession of such data, it must give a satisfactory explanation on how the party ended up in possession of such data.

Apart from the Cybercrimes Act, interception of communications is also criminalised under the RICA Act. Section 2 of the RICA Act provides that no person may intentionally intercept or attempt to intercept or authorise or procure any other person to intercept or to attempt at any place in the Republic, any communication in the course of its occurrence or transmission. It is argued that attempting to intercept or monitor a data communication unlawfully is as sanctionable as actually doing it is.^76^ However, lawful grounds of justification do apply, such as necessity, private defence, lawful interception, consent, court order or interception directive.^77^ It should be noted that the Constitutional Court declared large sections of the RICA Act unconstitutional, and the government was directed to amend the law.^78^

The Constitutional Court had to decide in “AmaBhungane” case whether mass surveillance and state actioned searches in terms of the RICA Act were constitutional. The court applied the two-stage analysis which involved considering whether a constitutional right had been infringed and secondly whether that infringement could be justified in terms of section 36 of the Constitution (the limitation clause). This is because the right to privacy is not an absolute right and can be limited if there are countervailing public interests or conflicting rights of others.^79^ In applying this test, the apex court held that the surveillance techniques adopted by the state infringed on section 14 of the Constitution of the Republic of South Africa (right to privacy). Upon application of the limitation clause, the Constitutional Court held that the RICA Act did not pass constitutional master because of the lack of adequate safeguards for independent judicial supervision and the notification of subjects of surveillance. It should also be noted that the RICA Act only applies to communications, but the scope of the Cybercrimes Act is much wider and covers not only intercepting communications but unlawful access to any form of data, computer program, computer storage medium and computer system.

#### Unlawful acts

To unlawfully access any data, criminals usually make use of hacking tools which can be software tools, hardware tools, passwords, and access codes. The Cybercrimes Act criminalises the intentional use and possession of any software or hardware tools for the purposes of unlawfully accessing, intercepting, and interfering with data.^80^ Section 4 of the Cybercrimes Act criminalises any activities where software or hardware tools have been used for unlawful access, unlawful interception, and unlawful interference. Closely related to possession of hacking tools is the offence of unlawfully and intentionally acquiring, possessing, providing to another person or using a password, an access code or similar data or device for purposes of contravening the provisions of section 2 (1) or (2), 3 (1), 5 (1), 6 (1), 8 or 9 (1) of the Cybercrimes Act.^81^ Password, access code or similar data or device includes a secret code or pin, an image, a security token, an access card, any device, biometric data or a word or a string of characters or numbers used for financial transactions or user authentication in order to access or use data, a computer program, a computer data storage medium or a computer system.^82^

Cronje et al. warn that ‘great caution and vigilance ought to be exercised by any person making use of digital technologies, considering that legally it is *prima facie* immaterial whether unlawful software or hardware tools in one’s possession are subject to unlawful use by another’.^83^ Considering the use of hacking tools and malware by cybercriminals to facilitate the commission of various crimes this particular offence should be considered as a deterrent and additional strategy to curb cybercrime.^84^

#### Unlawful interference

Section 5 of the Cybercrimes Act criminalises actions relating to interfering with data or computer programs. The Cybercrimes Act provides some guidance on what constitutes an ‘interference’.^85^ Actions such as deleting, altering, rendering vulnerable, damaging, deteriorating, rendering meaningless, useless or ineffective, obstructing, interrupting, or denying access to data or a computer program fall within the scope of ‘interference’.^86^ The fact that the legislature considered a tailored definition for the word ‘interference’ is likely to ensure legal certainty for the courts when dealing with cyber-interferences of various natures.^87^ Watney characterizes communications privacy as protection against interference and intrusion regarding communications that occur on websites visited, as well as electronic mails sent and received.^88^ Considering the value that data or programs have in the modern age, it is important to have an offence that provides legal protection measures to actions aimed at compromising data.^89^

Section 6 of the Cybercrimes Act focuses not on data or a computer program but on a computer data storage medium or a computer system. Like section 5, section 6 provides a clear definition for what constitutes an interference in this context. The Cybercrimes Act provides that where there is either a permanent or temporary alteration of any resource, an interruption or impairment to the functioning, confidentiality, integrity or availability of a computer data storage medium or a computer system, an interference in terms of section 6 has indeed occurred.^90^ It is therefore submitted that the legislature is likely to have considered the various eventualities of interference as tested by the courts and reported by law enforcement agencies, and accordingly resolved to provide a comprehensive definition for ‘interference’ in this particular context.

Unlawful activities such as website defacement fall within the ambit of sections 5 and 6 of the Cybercrimes Act. Website defacement occurs when an original content of a website is replaced by a cyber-attacker’s own content.^91^ Famous website defacement includes the Ashley Madison website which was defaced by hackers who went by the name Impact Team.^92^ Website defacement is commonly used to protest against social and political injustice around the globe, where hacktivists often rely on website defacement to spread their ideological messages to a wider audience.^93^ Recently, the State of Georgia was also a victim of a massive web defacement attack.^94^

### Malicious computer-related crimes

The crimes of cyber fraud, cyber forgery, uttering and extortion may be considered as malicious computer-related crimes.^95^

#### Cyber fraud

Fraud is a crime in terms of common law in South Africa. Fraud is defined as the unlawful and intentional making of a misrepresentation which causes actual prejudice, or which is potentially prejudicial to another.^96^ This form of cybercrime will usually be found in the form of “phising” and “spoofing”. The former activity entails sending out an e‑mail purporting to require information from the recipient for some legitimate purpose in the form of a request for personal details, e.g. for a bank account.^97^ Sometimes these messages direct the recipients to a “spoof” false website, usually a website controlled by the criminal purporting to be a legal website—again usually resembling that of a genuine bank. After having acquired the personal information, the identity thief can gain access to the victims through banking or shopping online channels and fraudulently purchase goods and services.^98^

South Africa has witnessed an increase in SMS fraud scams and SIM swap in the banking sector.^99^ In a recent court case, criminals were found guilty of SIM swap and unlawful interception of data.^100^ The criminals used software to record all keystrokes, including usernames and passwords and unlawfully obtained information required by mobile operators before a SIM swap could be processed on a particular cell phone. Once the SIM swap was processed, all bank notifications would be received by the criminal without the owner of the bank accounts being aware. Phishing scams are another top form of online fraud which is affecting a lot of people.^101^ Cassim defines phishing as ‘criminal acts that are carried out online to coerce victims to disclose personal or secretive information about themselves’ and both the business sector and the consumer are victims.^102^ Watney also provides the definition of phishing as an email fraud method in which the perpetrator sends out legitimate-looking emails in an attempt to gather personal and financial information from recipients.^103^

To address these new methods of committing fraud, the Cybercrimes Act makes provisions for a form of fraud which takes place by means of data and computer programs. Section 8 of the Cybercrimes Act provides that any person who unlawfully and with the intention to defraud makes a misrepresentation by means of data or a computer program or through any interference with data or a computer program or interference with a computer data storage medium or a computer system which causes actual or potential prejudice to another person is guilty of the offence of cyber fraud. While commenting on the now repealed sections 86 and 87 of the ECT Act, Cassim acknowledged that the ECT Act does not address the crime of phishing *per se* but argued that when offenders clone debit or credit cards they commit the offence of fraud.^104^

There have been arguments that there was no need for the Cybercrimes Act to deal with cyber fraud as it is a crime that could still be prosecuted under common law. Mabunda argued that a crime such as cyber fraud is not a true cybercrime and the mere presence of an internet element in the commission of a fraud, is not enough to elevate the crime to cyber fraud status.^105^ This argument stems from the transformational test that a true cybercrime is one which will cease to exist if we take away computers and the internet.

#### Cyber forgery and uttering

Cyber forgery involves the unlawful and intentional falsifying of data or a computer program to the actual or potential prejudice of another person.^106^ Cyber uttering is the unlawful and intentional passing off, of false data or a false computer program to the actual or potential prejudice of another person.^107^ There is a clear distinction between common law offences such as fraud, forgery and extortion in that the Cybercrimes Act links them directly to the use of data and a computer, or computer program.^108^ It is therefore assumed that the scope and application of the Cybercrimes Act in relation to these crimes *prima facie* seems sufficiently suited for the context of crimes committed in conjunction with data and/or computers.^109^ It is also assumed that the Cybercrimes Act, as a more comprehensive piece of legislation has cured possible deficiencies from the wording used in the ECT Act.

#### Cyber extortion

Any person who unlawfully and intentionally commits or threatens to commit any offence contemplated in section 3 (1), 5 (1), 6 (1) or 7 (1) (a) or (d) of the Cybercrimes Act for the purpose of obtaining any advantage from another person or compelling another person to perform or abstain from performing any act is guilty of the offence of cyber extortion.^110^ Ransomware attacks are good examples of cyber extortion crimes. A ransomware attack is an attack motivated by money as the criminals are interested in extorting money from their victims. South Africa’s state-owned company, Transnet, was a victim of a ransomware attack.^111^ Cybercriminals usually launch these cyber-attacks and ensure that the owners of a computer system do not have access to their system or data. The cybercriminal will then demand a ransom in exchange for giving the owners control over their system. The City of Johannesburg was also subjected to a ransomware attack and the criminals gained access to public facing data which resulted in the city suspending its online services.^112^ In September 2021, the Department of Justice was also hit by a ransomware attack.^113^ Most cybercriminals now demand ransom payment in cryptocurrencies, particularly Bitcoin.^114^

Watney explains that if a perpetrator hacks into a database and threatens to release the database in the public domain unless if he is getting paid, the hacker commits the offence of cyber extortion.^115^ If a person unlawfully infiltrates a computer program or data and demands the payment of a ransom in order to restore the computer system or in order not to share the data collected from the computer system, computer storage medium or computer network, the person will be committing the offense of cyber extortion. The penalty for cyber extortion is a sentence, as provided for in section 276 of the Criminal Procedure Act, that a court considers appropriate and within that court’s penal jurisdiction.^116^

#### Aggravated offences

Section 11 of the Cybercrimes Act provides for aggravated offences and maps out clearly that its application extends to sections 3 (1), 5 (1), 6 (1) or 7 (1) insofar as the passwords, access codes or similar data and devices are concerned.^117^ The guilt that can be assigned to an infringer/perpetrator in this context is strict in that where such a person knows or ought reasonably to have known/suspected that the computer system is restricted, the person may be found guilty of an aggravated offence. The Cybercrimes Act goes a step further by providing a succinct definition of a ‘restricted computer system’.^118^ A ‘restricted computer system’ is any data, computer program, computer data storage medium or computer system under the control of, or exclusively used by a financial institution and an organ of state as set out in section 239 of the Constitution,^119^ including a court and which is protected by security measures against unauthorised access or use.^120^

Accordingly, an unlawful interception of data, unlawful interference with data or a computer program, unlawful interference with a computer data storage medium or computer system, or unlawful acquisition, possession, provision, receipt or use of a password, access code or similar data or device of a financial institution (i.e., one of the main banking institutions) or a Government Ministry, results in an aggravated offence charged against the cybercriminal.^121^

#### Theft of incorporeal property

Section 12 of the Cybercrimes Act is a rather straightforward and easy-to-comprehend provision as it holds that ‘the common law offence of theft must be interpreted so as not to exclude the theft of incorporeal property’. Snail submitted that the court in *Van Heerden v S*^122^ affirmed the definition of theft as:

‘A person commits theft if he unlawfully and intentionally appropriates moveable, corporeal property which (a) belongs to, and is in the possession of another, (b) belongs to another but is in the perpetrator’s own possession; or (c) belongs to the perpetrator but is in another’s possession and such other person has a right to possess it which legally prevails against the perpetrator’s own right of possession provided that the intention to appropriate the property includes an intention permanently to deprive the person entitled to the possession of the property, of such property.’^123^

With the common law of theft, there must be an appropriation and intention to permanently deprive—however, it should be noted that this is difficult to prove when it comes to theft of information.^124^

### Malicious communications

#### Incitement of threats of damage to property or violence

Part II of chapter 2 of the Cybercrimes Act deals with malicious communications. There are three types of offences which are prescribed under this section. The Cybercrimes Act criminalises any forms of incitement of violence or threats to damage property. It is an offence for a person to disclose by means of an electronic communications service, a data message to a person, group of persons or the general public with the intention to incite the causing of any damage to property belonging to, or violence against a person or group of persons.^125^ It is also an offence to unlawfully and intentionally disclose a data message which threatens a person or group of persons with damage to property or violence.^126^ The section further provides that a reasonable person in possession of the same information would perceive the data message, either by itself or in conjunction with any other data message or information, as a threat of damage to property or violence to a person or group of persons. The Cybercrimes Act defines ‘damage to property’ as damage to any corporal or incorporeal property. The term ‘group of persons’ is defined as characteristics that identify an individual as a member of a group, which characteristics include without limitation, race, gender, sex, pregnancy, marital status, ethnic or social origin, colour, sexual orientation, age, disability, religion, conscience, belief, culture, language, birth, or nationality. It also defines violence as bodily harm.^127^

#### Revenge pornography

Section 16 of the Cybercrimes Act deals with revenge pornography or non-consensual pornography. Musoni relies on Bloom’s characterisation of revenge porn to define it as ‘non-consensual pornography/involuntary pornography, involves the distribution of sexually graphic images of an individual where at least one of the individuals depicted did not consent to the dissemination.’^128^ Section 16 of the Cybercrimes Act provides that ‘any person (“A”) who unlawfully and intentionally discloses, by means of an electronic communications service, a data message of an intimate image of a person (“B”), *without the consent* of B, is guilty of an offence’ (authors’ emphasis). Musoni states that the consent requirement is quite significant as it distinguishes pornography from non-consensual pornography. In the absence of consent, Musoni argues, any processing of intimate data by a third party can be a section 16 offence.^129^ The Cybercrimes Act specifically sets the parameters within which an intimate image can be perceived. This provision stretches far enough to include real and simulated images and specifies that where the image of a person is depicted nude, or where the bare or covered genital organs or anal region, the breast area of a female, transgender or intersex person are depicted.^130^

One of the criticism levelled against the revenge porn provision of the Cybercrimes Act is that criminal consequences are only against the original perpetrator who first disseminates the sexually graphic images, and there are no real consequences for any subsequent sharing by third parties.^131^ Snail argued that where the Cybercrimes Act comes short, the Protection of Personal Information Act^132^ (POPIA) can certainly create offences.^133^ POPIA provides minimum conditions or requirements which must be complied with when processing personal information. It defines personal information as information relating to an identifiable, living, natural person.^134^ A graphic image of a person is indeed personal information in terms of which legal consequences may result for third parties who process such images in accordance with the definition for processing.^135^

#### Aiding and abetting

Part III of chapter 2 of the Cybercrimes Act also criminalises anyone who unlawfully and intentionally attempts, conspires with any other person, or aids, abets, induces, incites, instigates, instructs, commands, or procures another person to commit an offence under the Cybercrimes Act.^136^

## Provisions which have not yet commenced

As mentioned earlier, there are a few sections of the Cybercrimes Act which have not come into operation yet. One of the reasons why these sections are not yet operational may be because there is preparatory work which needs to be done before these sections can be enforced. For example, for foreign states to rely on the provisions of the Cybercrimes Act to obtain assistance from South African police, they need to submit the request via the point of contact. At present, the point of contact is yet to be established.

Part VI of chapter 2 is yet to come into operation. Snail summarises that Part VI deals with the issuing of protection orders against suspected cyber harassment, cyber threats of damage to property or anyone inciting others to damage property, and revenge porn.^137^ Section 38 (1) (d), (e) and (f) of the Cybercrimes Act provides for any person who unlawfully or intentionally gives false information under oath or by way of affirmation knowing it to be false or not knowing it to be true, with the result that an expedited preservation of data direction contemplated in section 41 is issued; or a preservation of evidence direction contemplated in section 42 is issued; or a disclosure of data direction contemplated in section 44 is issued, is guilty of an offence and is liable on conviction to a fine or to imprisonment for a period not exceeding 2 years or to both such fine and imprisonment.^138^

In terms of section 40 (3) of the Cybercrimes Act, an Electronic Communications Service Provider (ECSP) may be required to comply with a real-time communication-related direction in terms of which the ECSP is directed to provide real-time communication-related information in respect of a customer, on an ongoing basis, as it becomes available. The ECSP must also comply with other directions such as the expedited preservation of data direction contemplated in section 41, a preservation of evidence direction contemplated in section 42, a disclosure of data direction contemplated in section 44 and any order of a designated judge. Section 40 (4) of the Cybercrimes Act provides that any indirect communication which is to be intercepted or any real-time communication-related information or traffic data which is to be obtained, at the request of an authority, court or tribunal exercising jurisdiction in a foreign state must further be dealt with in the manner provided for in an order referred to in section 48 (6), which is issued by the designated judge.

The non-commencement also applies to the direction for expedited preservation of data as contemplated in section 41 of the Cybercrimes Act. An ECSP, a financial institution or any person may be served an expedited preservation of data direction by a police official. The direction may relate to the preservation of data in its current status, not to deal in any manner with the data or to deal with data in a certain manner.^139^ Section 42 of the Cybercrimes Act deals with preservation of evidence direction which is issued by a magistrate or judge of the High Court upon written application by a police official. The preservation of evidence direction is valid for a period of 90 days from the time of service of the direction. A person, ECSP or financial institution who has been served with the direction may be directed to preserve an article in order to preserve its availability or integrity.^140^ Section 43 provides for instances where a police official may approach a magistrate or judge of the High Court and orally apply for preservation of evidence direction.

In terms of section 44 of the Cybercrimes Act, a police official may apply to a magistrate or judge of the High Court for the issuing of a disclosure of data direction. This disclosure of data direction will be used to obtain data which is subject to preservation in terms of an expedited preservation of data direction or a preservation of evidence direction or data held in a computer system or computer storage medium or available to a computer system.^141^

The date of commencement of chapter 5 of the Cybercrimes Act is also yet to be proclaimed. Chapter 5 allows for cooperation in the preservation of evidence where a crime has been committed in a foreign country.^142^ The chapter also allows for the National Commissioner of Police or the National Head of the Directorate to assist a law enforcement agency of a foreign state by providing any information obtained in any investigation of an offence.^143^ SAPS may also receive any information from a foreign state, subject to such conditions regarding confidentiality and limitation of use as may be agreed upon, which may assist SAPS in initiating an investigation or lead to further cooperation with a foreign state to carry out an investigation regarding the commission or suspected commission of an offence.^144^ For a foreign state to obtain assistance and cooperation from South Africa, there is a process with checks and balances, as provided in chapter 5, which must be followed.

Chapter 6 of the Cybercrimes Act deals with the designated point of contact. Section 52 of the Cybercrimes Act provides for the establishment of an office within the existing structures of the SAPS to be known as the designated point of contact for the Republic. The National Commissioner is also required to equip, operate, and maintain the designated point of contact. Unlike the Council of Europe Convention on Cybercrime (Budapest Convention) where the point of contact is operational for 24 h, 7 days a week^145^, the Cybercrimes Act is silent on this. Cassim had previously recommended that South Africa needs to revise some procedural provisions to comply with the Budapest Convention such as introducing a 24/7 contact centre.^146^ The Cybercrimes Act only indicates that the point of contact must ensure the provision of ‘immediate assistance’ for the purpose of proceedings or investigations regarding the commission of an offence in a foreign state.^147^

Section 54 of the Cybercrime Act which provides that an ECSP must, within 72 h of having become aware, report an offence committed in terms of Part I of the Cybercrimes Act to the SAPS will also not commence. The remainder of the Cybercrimes Act will apply save for the exclusion of section 11B, 11C, 11D and Section 56A (3) (c)–(e) of the Criminal Law (Sexual Offences and Related matter Amendment Act, Act 32 of 2007) in Chapter 9 in the schedule of Laws repealed by Section 58 of the Cyber Crimes Act.

## Conclusion

South Africa is no longer a safe haven for cybercriminals. The Cybercrimes Act is a welcome development in South Africa’s criminal law jurisprudence. Not only does the Cybercrimes Act prescribe certain conduct as criminal offences, it also empowers South African courts to adjudicate offences (chapter 3). South African law enforcement agents are also empowered to exercise jurisdiction to search and seize evidence (chapter 4). It should be noted that while the law generally permits the exercise of jurisdiction, law enforcement agents will continue facing jurisdictional challenges when prosecuting cybercrime.^148^ This is quite evident in instances where law enforcement agents seek to access, seize and search remotely based cloud evidence. Chapter 5 of the Cybercrimes Act provides a solution to jurisdictional hurdles as it allows for cooperation between foreign countries and domestic law enforcement agents. Chapter 6 also establishes the point of contact where domestic and foreign law enforcement agents can cooperate in cybercrime investigations. It is important that the legislature makes sure that these chapters come into commencement as a matter of urgency.

It is also important for South Africa to ensure that legal practitioners and adjudicators (magistrates and judges) receive education on cybercrime, gathering of electronic evidence and admissibility of digital evidence during court processes.^149^ While it is true that South Africa lacks skilled experts to assist in cybercrime investigations and gathering of evidence, there should be efforts in improving the work of Specialised Commercial Crimes Courts. Specialised Commercial Crimes Courts have been tackling various commercial crimes through a system of magistrates, prosecutors and other court officials specifically dedicated to the task.^150^ Since police officers are mainly responsible for initial investigations of cybercrime, it is prudent for them to also undergo training to learn new skills around gathering of electronic evidence and forensics.

## Footnotes

1 The Cybercrimes Act 19 of 2020.

2 Francis Cronje, Sizwe Snail, Anthony Olivier and Jason Jordaan, *The Cybercrime Act—What you need to know* (2022) at 3–4.

3 At the time of writing of this paper, the commencement dates for part VI of chapter 2, section 38 (1) (d) and (e), section 40 (3) and (4), sections 41, 42, 43, 44, chapter 5 (mutual assistance), and chapter 6 (designated point of contact) are yet to be proclaimed.

4 The Electronic Communications and Transactions Act 25 of 2002.

5 Sizwe Snail, ‘Çybercrime in South Africa—hacking, cracking and other unlawful online activities’ 2009 *Journal of Information, Law & Technology (JILT)* 1 8 at 63.

6 Cronje et al. op cit note 4 at 2.

7 Watney, Murdoch ‘Cybercrime and the investigation of cybercrime’ in Papadopoulos S & Snail S *Cyberlaw @SAIII: The law of the internet in South Africa* 3 (ed) at 337.

8 Cronje et al. op cite note 4 at 4.

9 Ibid at 5.

10 Cronje et al. op cit note 4 at 5.

11 *S v Berend Howard* unreported case no 41/258/02, Johannesburg Regional Magistrates Court.

12 Sizwe Snail ‘Cyber crime under the ECT Act’ 2008 *Juta’s Business Law* 63 at 63.

13 For an accused to be found guilty of an offence and sentenced, the type of conduct with which he is charged must have been clearly recognised by the law as a crime, before the conduct took place and without the court having to stretch the meaning of the words and concepts in the definition to bring the particular conduct of the accused within the compass of the definition. CR Snyman *Criminal Law* 5 (ed) (2008) at 36.

14 Snyman ibid at 38.

15 Snyman op cit note 15 at 39.

16 Ibid.

17 Every accused person has a right to a fair trial, which includes the right to adduce and challenge evidence. The Constitution of the Republic of South Africa, 1996.

18 Snyman op cit note 15 at 44.

19 Snyman op cit note 15 at 45.

20 Ibid.

21 Fawzia Cassim ‘Addressing the challenges posed by cybercrime: A South African perspective’ *2010 Journal of International Commercial Law and Technology *118 at 118.

22 Cronje et al. op cit note 4. Please also see Garg, N. ‘Towards the Impact of Hacking on Cyber Security’ 61 (2018) *IIOAB Journal* 9.

23 Sizwe Snail ‘The convergence of legislation on cybercrime and data protection in South Africa: A practical approach to the Cybercrimes Act 19 of 2020 and the Protection of Personal Information Act 4 of 2013’ 2022 *Obiter Law Journal* 536 at 544.

24 Van der Merwe et al. *Information Communication Technology Law 3* (2022) at 88 also see the cases of S v Ntombikayise (SCCC 181/2004) and S v Englbrecht, Easypayroll and Lambrecht (SCCC 111/05).

25 *R v Douvenga* (District Court of the Northern Transvaal, Pretoria, case no 111/150/2003, 19 August 2002, unreported).

26 *Okundu v S *[2016] ZAECGHC 131.

27 *Mgoqi v S *[2020] ZAECGHC 33.

28 *Ibid.*

29 *Salzmann v S* (755/18) [2019] ZASCA 145; [2020] 1 All SA 361 (SCA); 2020 (2) SACR 200 (SCA).

30 Cronje et al. op cit note 4 at 6.

31 Maat,* Cybercrime: A comparative Analysis Law Analysis *(LLM Thesis, Unisa, 2004) ft 145.

32 Njabulo Mthimunye, ‘The intersection between Cyber Crimes laws and Data Protection laws in South Africa’ (2020) iAfrikan online article (accessed at https://www.iafrikan.com/2020/07/17/the-intersection-between-Cyber Crimes-laws-and-data-protection-laws-in-south-africa/ on 22/9/20).

33 Fawzia Cassim ‘Addressing the spectre of phishing: are adequate measures in place to protect victims of phishing?’ 2014 *Comparative and International Law Journal of Southern Africa* 401 at 415.

34 Cassim op cit note 23 at 128.

35 Section 19 of the Cybercrimes Act.

36 Cassim op cit note 23 at 138.

37 African Union Convention on Cyber Security and Personal Data Protection, 27 June 2014, (Ex.CL/846(XXV)) accessed at https://au.int/en/treaties/african-union-convention-cyber-security-and-personal-data-protection.

38 Article 25 (1) of the African Union Convention on Cyber Security and Personal Data Protection.

39 Matanzima and Snail ka Mtuze, S (2014) ‘Cyber security in Africa—A Cyber Law framework’ in *Without Prejudice*, Vol. 14 accessed at http://withoutprejudice.co.za/pdf/WP-October20”14.pdf on 29/3/2023.

40 Snail op cit note 25 at 545.

41 Ibid.

42 Ibid.

43 Bert-Jaap Koops, ‘The internet and its opportunities for cybercrime’ (2011) 9 *Tilburg Law School Legal Studies Research Paper Series* 735 at 739.

44 Sagwadi Mabunda, ‘Is it cyberfraud or good ol’offline fraud: A look at section 8 of the South African Cybercrimes Bill’ (2018) 2 *Journal of Anti-Corruption Law* 58 at 61.

45 Salami fraud occurs when a tiny, unnoticeable fraction of many different bank accounts is sliced off. Jonathan Clough, ‘The Council of Europe Convention on Cybercrime: Defining “Crime” in a digital world’ (2012) 13 *Criminal Law Forum* 363 at 372.

46 Ibid.

47 David Wall, ‘The internet as a conduit for criminal activity’ in Pattavina, A *Information Technology and the Criminal Justice System* (2015) at 81.

48 Mark N. Gasson and Bert-Jaap Koops, ‘Attacking Human Implants: A New Generation of Cybercrime’ (2013) 5 *Law, Innovation & Technology *248 at 250.

49 Wall op cit note 53 at 82.

50 Gasson and Koops op cit note 54 at 250.

51 Clough op cit note 51 at 371.

52 Wall op cit note 53 at 82–83.

53 Clough op cit note 51 at 371.

54 Susan W Brenner, ‘At light speed: Attribution and response to cybercrime/terrorism/warfare’ (2007) 97 *The Journal of Criminal Law & Criminology* 379 at 385.

55 Section 2 (1) of the Cybercrimes Act.

56 Section 2 (2) (a) of the Cybercrimes Act.

57 Section 2 (2) (b) (i) of the Cybercrimes Act.

58 Section 2 (2) (b) (ii) of the Cybercrimes Act.

59 Section 2 (2) (c) (i) of the Cybercrimes Act.

60 Section 2 (2) (c) (ii) of the Cybercrimes Act.

61 Fernando M Pinguelo and Bradford W Muller, ‘Virtual crimes, real damages: A primer on cybercrimes in the United States and efforts to combat cybercriminals’ (2011) 16 *Virginia Journal of Law and Technology* 116 at 132.

62 Ibid.

63 Snail op cite note 25 at 537.

64 Ibid.

65 See Dinan “Ware-Withal: EBlaster the ultimate tool of the spies who love you” 2003 Boston Business Journal https://www.bizjournals.com/boston/blog/mass-high-tech/2003/06/ware-withal-eblaster-the-ultimate-tool-of.html (accessed 2021-07-13): “*Sign on for eBlaster and you’ll get hourly reports detailing every keystroke typed by your kids, husband, wife, sweetheart or employees. You’ll be able to read both sides of their e‑mail conversations via Hotmail, Yahoo, AOL, Microsoft Outlook and EarthLink. Both sides of all instant messages and back-and-forth from inside chat rooms is reported in detail, including chat with providers AOL and MSN Instant Messenger*”.

66 Snail op cit note 25 at 548.

67 Ibid.

68 Ibid.

69 Regulation of Interception of Communications and Provision of Communication-Related Information Act 70 of 2002.

70 Snail op cit note 25 at 548.

71 RICA Act, section 1.

72 Section 3 (1) of the Cybercrimes Act.

73 *Okundu v S* (CA&R117/16) [2016] ZAECGHC 131 (22 November 2016).

74 Section 3 (2) of the Cybercrimes Act.

75 Section 3 (3) of the Cybercrimes Act.

76 Papadopolous and Snail op cit note 25 at 65. Reinhardt Buys, ‘Love Hurts’ (2000) *De Rebus* 33.

77 Ibid.

78 *AmaBhungane Centre for Investigative Journalism NPC v Minister of Justice and Correctional Services; Minister of Police v AmaBhungane Centre for Investigative Journalism NPC* [2021] ZACC 3; 2021 (4) BCLR 349 (CC); 2021 (3) SA 246 (CC).

79 Konrad Lachmayer and Norman Witzleb, ‘The challenge to privacy from ever increasing state surveillance: A comparative perspective’ (2014) *University of New South Wales Law Journal* 748 at 751.

80 Section 4 Cybercrimes Act.

81 Section 7 (1) Cybercrimes Act.

82 Section 7 (3) Cybercrimes Act.

83 Snail op cit note 25 at 548.

84 Cronje et al., *The Cybercrime Act—What you need to know* (2022) at 67.

85 Snail op cit note 25 at 549.

86 Section 5 (2) of the Cybercrimes Act.

87 Snail op cit note 25 at 549.

88 Murdoch Watney, ‘The Evolution of Internet Legal Regulation in Addressing Crime and Terrorism’ 2007 *The Journal of Digital Forensics, Security and Law* 40 at 49.

89 Cronje et al. op cit note 90 at 67.

90 Section 6 (2) of the Cybercrimes Act.

91 C. Jordan Howell, George W. Burruss, David Maimon & Shradha Sahani, ‘Website defacement and routine activities: considering the importance of hackers’ valuations of potential targets’ (2019) *Journal of Crime and Justice* 1 at 2.

92 Ibid.

93 Ibid.

94 More than 15,000 websites were defaced, and 2000 websites were offline for a couple of hours. The content of the websites was replaced with a photo of the former President of Georgia, Mikheil Saakashvili, with the words “I’ll be back”. ‘Georgia hit by massive cyber-attack’ BBC News Available at https://www.bbc.com/news/technology-50207192 accessed on 29 March 2023.

95 Snail op cit note 25 at 549.

96 Mabunda op cit note 50 at 60.

97 Van der Merwe op cit note 26 at 80.

98 Ibid.

99 Cassim discusses the SMS fraud case where a Vodacom employee was working with a syndicate to intercept SMS notifications from banks to their customers. This scam resulted in Rand 7 million being siphoned off from customers’ accounts. Another case related to a fraud happened in the Mpumalanga Education Department in which huge amounts of money were paid into an unsuspecting woman’s account from the Department’s bank. Cassim op cit note 18 at 130–2.

100 *S v Myeni *2019 (1) SACR 360 (ECG) para 10.

101 Cassim op cit note 23 at 403.

102 Ibid.

103 Watney op cit note 39 at 344.

104 Cassim op cit note 23 at 415.

105 Mabunda op cit note 50 at 58.

106 Section 9 (1) of the Cybercrimes Act.

107 Section 9 (2) of the Cybercrimes Act.

108 For example, in Van *Heerden v S* (A160/2016) [2016] ZAFSHC 191, the Court gave the Common Law definition of fraud by stating that, “*Fraud is the unlawful and intentional making of a misrepresentation which causes actual prejudice or which is potentially prejudicial to another*.” See *Hattingh v S* (A307/2015) [2016] ZAWCHC 199 in respect of the Common Law definition of forgery; *Ndlovu v S* (CA&R14/2016) 2016 ZAECBHC 12 for a characterization of the Common Law crime of extortion; and *Cossie v S* (A114/2011) [2011] ZAFSHC 169 in respect of the Common Law crime of uttering.

109 Snail op cit note 25 at 550.

110 Section 10 of the Cybercrimes Act.

111 Ryan Gallagher and Paul Burkhardt, ‘Death kitty ransomware linked to South African port attack’ 29 July 2021 Bloomberg. Available at https://www.bloomberg.com/news/articles/2021-07-29/-death-kitty-ransomware-linked-to-attack-on-south-african-ports, accessed on 14 September 2021.

112 Shortly after the City of Johannesburg was subjected to ransomware attacks, most South African banks were also subjected to a similar attack (South African Banking Risk Information Centre (SABRIC) report). The City of Johannesburg attacks started with a ransom note which was delivered via email to both unattended as well as staff e‑mail addresses, all of which were publicly available. Malibongwe Dayimani, Soyiso Maliti and Genevieve Quintal, ‘SA now hostage to cyber ransom’ Sunday Dispatch 26 October 2019 page 5. In July 2019, City Power came under a ransomware attack that prevented thousands of prepaid customers from buying electricity. Shaun Smillie, ‘Hackers hold city, banks to ‘ransom’ Saturday Star 26 October 2019.

113 ‘Update on progress in restoring justice services following ransomware attack’. Department of Justice Media Statement 21 September 2021. https://www.justice.gov.za/m_statements/2021/20210921-IT-Systems-RestorationProgress.pdf accessed 19 January 2022.

114 In the case of the City of Johannesburg, the criminals who went by the name Shadow Kill Hackers demanded payment of 4 Bitcoin which was equivalent to half a billion Rand at the time. Catalin Cimpanu, ‘City of Johannesburg held for ransom by hacker gang’ NET innovation 25 October 2019. Available at https://www.zdnet.com/article/city-of-johannesburg-held-for-ransom-by-hacker-gang/ accessed on 14 October 2022. Criminals also attacked a petroleum company in Mexico. On 10 November 2019, a state-owned oil company in Mexico, Petroleos Mexicanos (Pemex) was attacked by cybercriminals who were demanding 565 Bitcoin ($ 4.9 Mio.). The cybercriminals claimed to have gathered sensitive data from the Pemex network. Ionut Arghire, ‘Mexican oil company Pemex hit by ransomware’ Security Week 12 November 2019. Available at https://www.securityweek.com/mexican-oil-company-pemex-hit-ransomware accessed on 14 October 2022.

115 Watney op cit note 9 at 344.

116 Section 19 (4) Cybercrimes Act.

117 Snail, ‘The convergence’ at 550.

118 Murdoch Watney, ‘Cybercrime and the investigation of cybercrime’ in Papadopoulos S & Snail S *Cyberlaw @SA IV: The law of the internet in South Africa* 4 ed at 490.

119 Section 1 of the Cybercrimes Act defines financial institutions in the following manner: “means a financial institution as defined in section 1 of the Financial Sector Regulation Act, 2017 (Act No. 9 of 2017)”. S1 of the Financial Sector Regulation Act 9 of 2017 defines a financial institution as any of the following, other than a representative: (a) A financial product provider; (b) a financial service provider; (c) a market infrastructure; (d) a holding company of a financial conglomerate; or (e) a person licensed or required to be licensed in terms of a financial sector law. Section 239 of the Constitution provides that: “organ of state” means—(a) any department of state or administration in the national, provincial or local sphere of government; or (b) any other functionary or institution—(i) exercising a power or performing a function in terms of the Constitution or a provincial constitution; or (ii) exercising a public power or performing a public function in terms of any legislation, but does not include a court or a judicial officer”.

120 Section 11 (1) (b) of the Cybercrimes Act.

121 Watney op cit note 124 at 490.

122 *Van Heerden v S* (A160/2016) [2016] ZAFSHC 191 par 6, where the Court cited Snyman *Criminal Law* (2008) LexisNexis 483.

123 Snail op cit note 26 at 551.

124 The UK case of *Oxford v Moss* [1978] 68 Cr App Rep 183 is a good illustration of the limitations in applying traditional laws to cybercrimes. In this case, the question was whether the accused had committed the crime of theft. A student accessed an examination paper and read it in advance of an exam. He was charged with the offence of theft. The court held that there could be no theft of information. The Supreme Court had to consider whether confidential information could be the object of the theft and whether the appropriation of the information could amount to fraud.

125 Section 14 of the Cybercrimes Act.

126 Section 15 of the Cybercrimes Act.

127 Section 13 of the Cybercrimes Act.

128 Melody Musoni, ‘The Criminalisation of ‘Revenge Porn’ in South Africa’ (2019) *Obiter Law Journal* 61 at 62.

129 Ibid at 70.

130 Section 16 (2) (b) of the Cybercrimes Act.

131 Musoni op cit 134 at 71.

132 Protection of Personal Information Act 4 of 2013.

133 Snail op cit note 25 at 552.

134 Section 1 of POPIA.

135 Snail op cit note 25 at 553.

136 Section 17 of the Cybercrimes Act.

137 Snail op cit note 25 at 545.

138 Cronje et al. op cit note 90 at 5.

139 Section 41 (4) of the Cybercrimes Act.

140 Section 42 (3) (a) of the Cybercrimes Act.

141 Section 44 (1) (a) of the Cybercrimes Act.

142 Section 46 of the Cybercrimes Act.

143 Section 47 (1) of the Cybercrimes Act.

144 Section 47 (2) of the Cybercrimes Act.

145 Article 35 of the Budapest Convention.

146 Cassim op cit note 23 at 423.

147 Section 52 (3) of the Cybercrimes Act.

148 Cronje et al. op cit note 4 at 9.

149 Ibid at 11.

150 Snail op cit note 25 at 544.
